# Supplementary material for: PARylation in Parkinson’s disease: a bridge between Lewy body formation and neuronal cell death
Source: Cell Death Dis. 2026 Jun 23;17(1):776. doi: 10.1038/s41419-026-08880-1 (PMC13538525; doi:10.1038/s41419-026-08880-1)
Supplement: Supplementary file 1 [file 41419_2026_8880_MOESM1_ESM.pdf]

Supplementary Figures 1-6

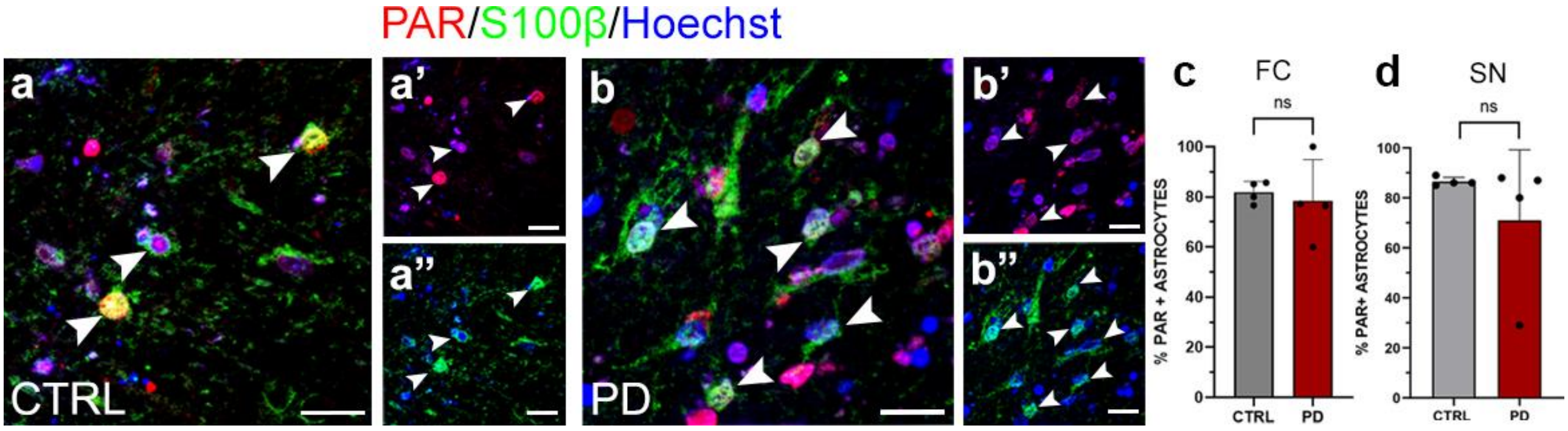

Supplementary Figure 1. Confocal analysis of PAR in astrocytes. (**a-b''**) representative image showing PAR nuclear distribution (white arrowheads) in astrocytes (S100β positive) of *substantia nigra* of CTRL subjects (**a-a''**) and PD patients (**b-b''**). Scale bar, 20 µm. The graphs show the percentage of PAR positive astrocytes nuclei in frontal cortex (**c**, FC: CTRL, 132 astrocytes, n = 4 vs PD, 171 astrocytes, n = 4) and *substantia nigra* (**d**, SN: CTRL, 141 astrocytes, n = 4 vs PD, 150 astrocytes n = 4). Data are reported as mean ± standard deviation. Mann-Whitney test, p>0.05; ns. CTRL = control; FC = frontal cortex; N = nucleus; ns = non-significant; SN = *substantia nigra*.

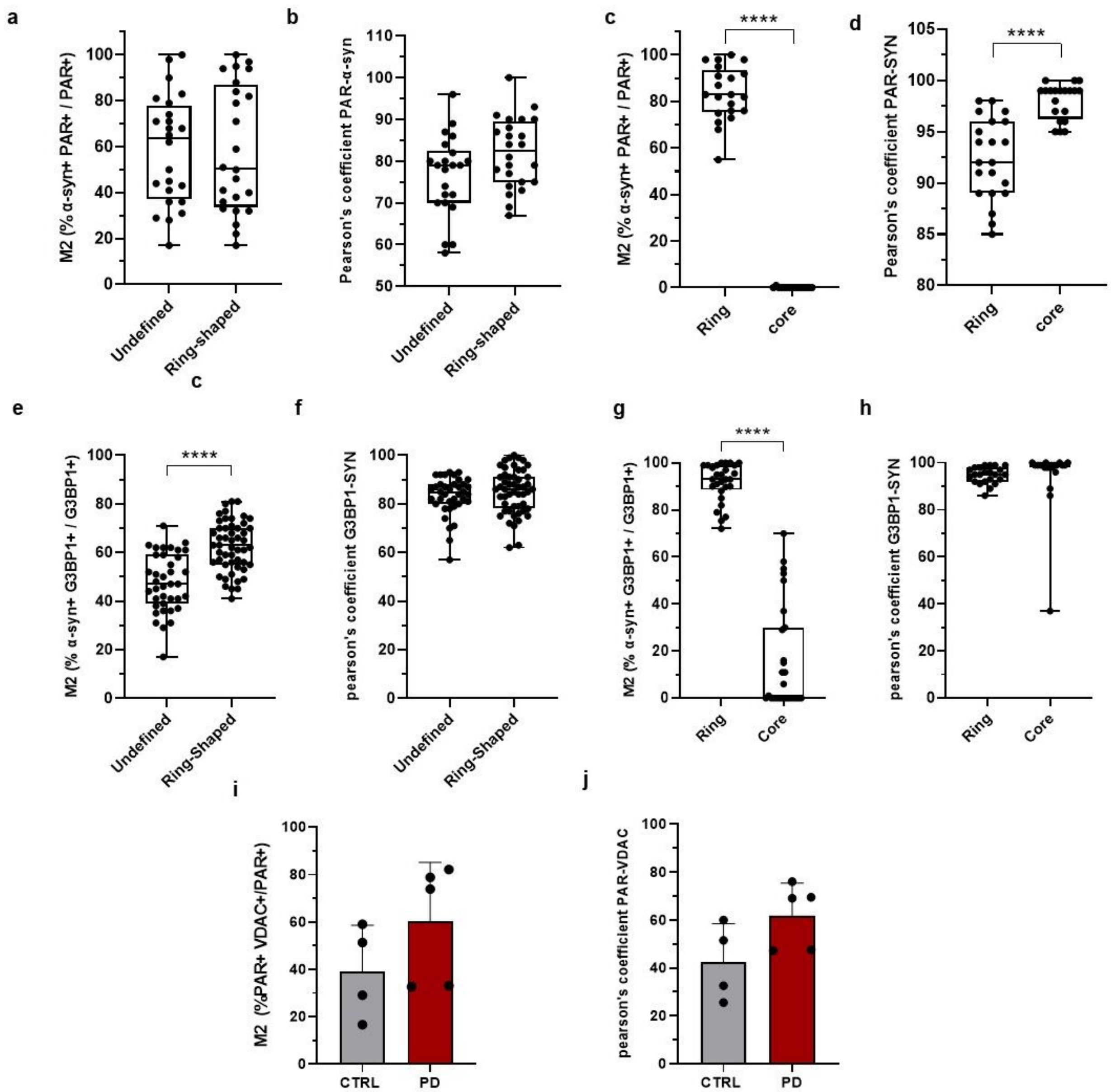

Supplementary Figure 2. Colocalization of PAR/G3BP1 with α-synuclein and PAR/VDAC1. M2 Manders (**a**, **c**, **e**, **g**, **i**) and Pearson's coefficients (**b**, **d**, **f**, **h, j**) for PAR and G3BP1 colocalization with α-synuclein in undefined compared to ring shaped aggregates (**a**, **b**, **e**, **f**) or in the different part of a mature LB (ring and core; **c**, **d**, **g**, **h**) and PAR and VDAC1 (**i**, **j**). (**c**, **d**, **g**, **h**) in the LB Manders coefficient (M2) and Pearson's coefficient are opposite in the core region due to their intrinsic meaning: M2 considers the colocalization related to the total signal of PAR or G3BP1, while Pearson high level in the core represents the consistent smooth signal of both the antigens (α-synuclein and PAR/G3BP1) in the core LB region.

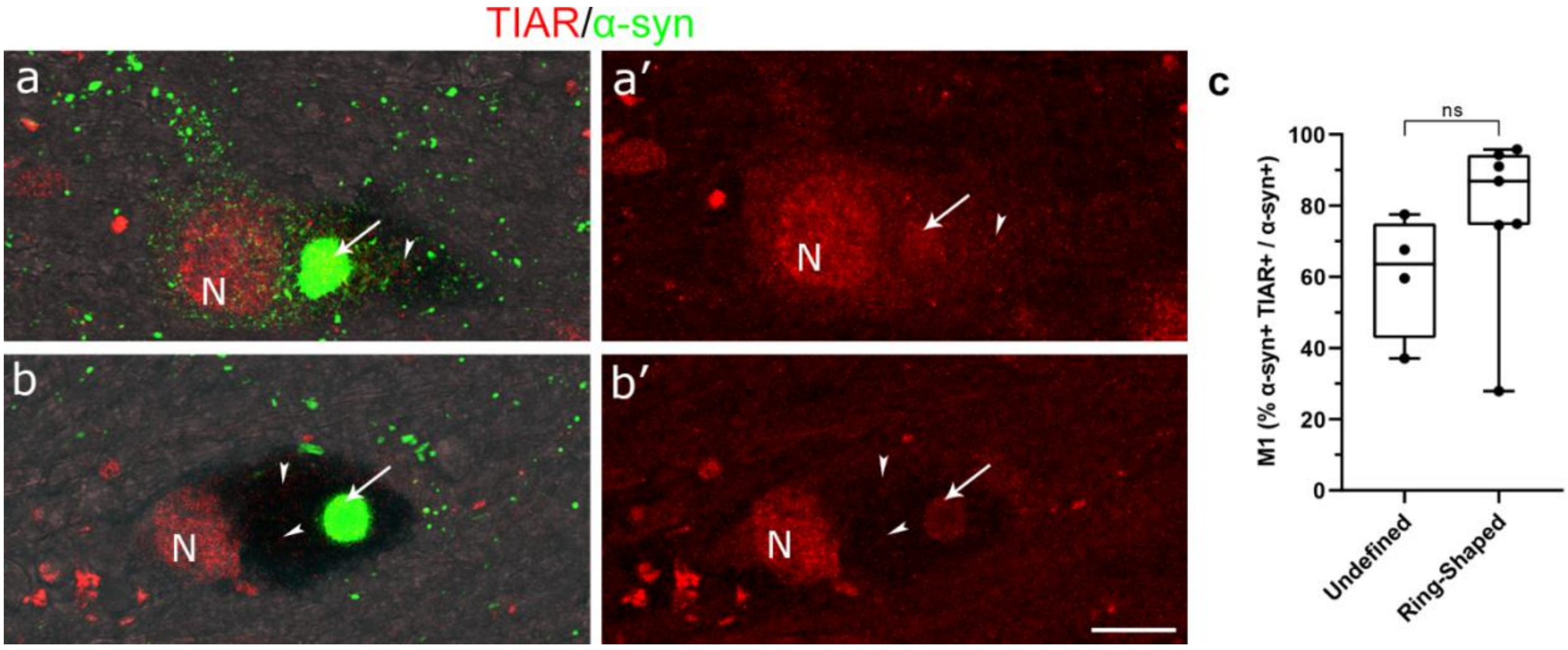

Supplementary Figure 3. Confocal analyses of TIAR and α-synuclein during LB morphogenesis in *substantia nigra* of PD patients. (**a, a'**) TIAR localizes in the nuclei and shows a point-like signal in the cytoplasm (white arrowhead) between the α-synuclein network and neuromelanin granules, visible with phase contrast, and colocalizing with α-synuclein undefined aggregate (white arrow). (**b, b'**) When the ring-shaped structure is formed TIAR is present in both the nucleus and cytoplasm (white arrowheads) where it colocalizes with α-synuclein (white arrow). Colocalization increases from the undefined to the ring-shaped aggregate. Scale bar, 20 µm. The graph in **c** (n= 1 PD, undefined = 4; ring-shaped = 7) indicates the percentage of α-synuclein colocalizing with TIAR expressed by Mander's coefficient (M1). Data are reported as mean ± standard deviation. Mann-Whitney test, ns. N = Nucleus. ns = non-significant.

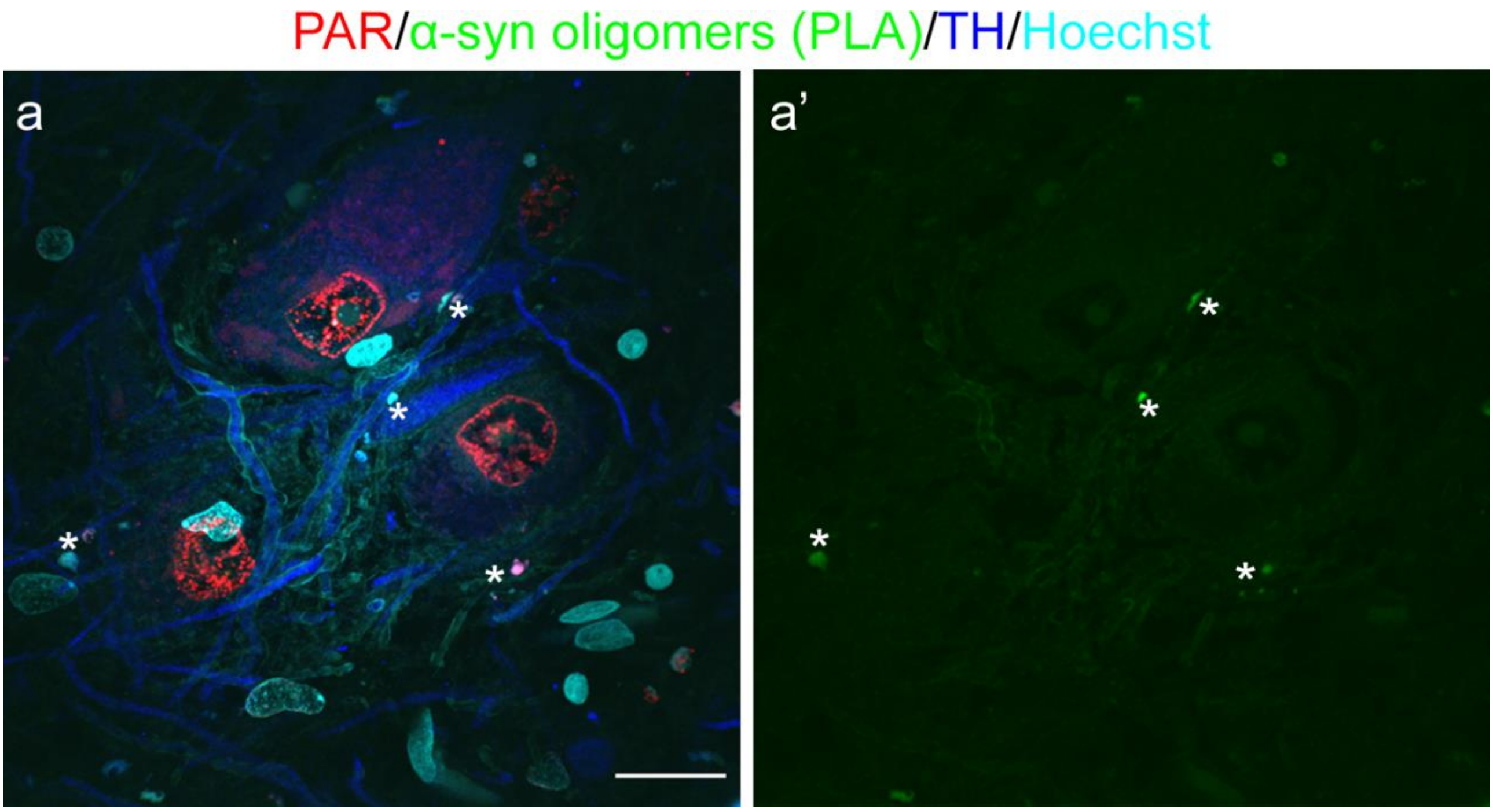

Supplementary Figure 4 PAR and TH immunostaining and α-synuclein oligomer PLA signal in CTRL subjects. a PLA signal (green) is absent in *substantia nigra* neurons (TH-positive, blue). PAR localize in the nucleus (red)**. a'** green channel showing the absence of PLA puncta, white asterisks indicate tissue autofluorescence. Nuclei are counterstained using Hoechst (cyan). Scale bar, 20 μm.

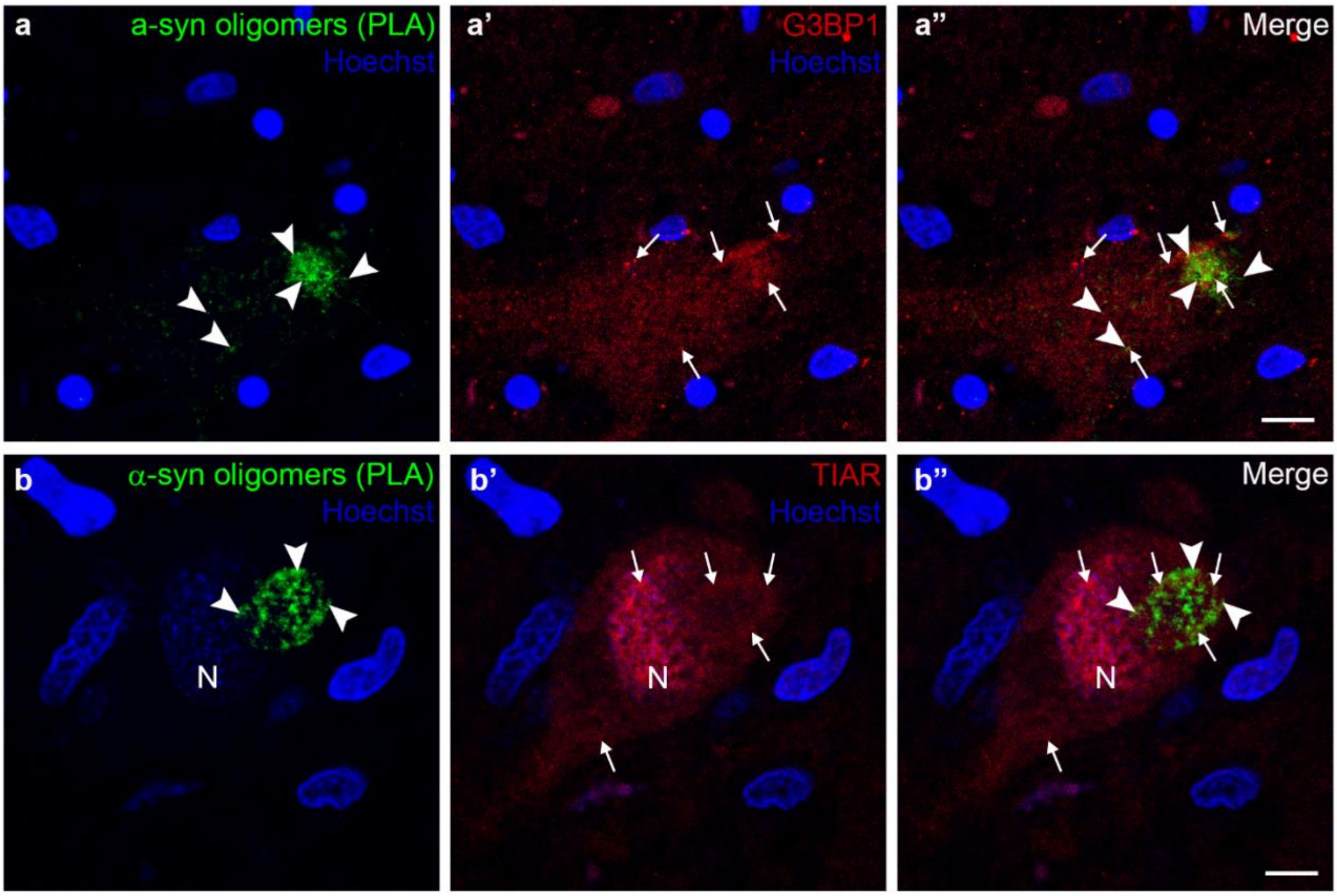

Supplementary Figure 5. Stress granules and α-synuclein oligomers. α-Synuclein oligomers are diffused in the cytoplasm and in an undefined aggregate (**a, b**; arrowheads); G3BP1 (**a'**) and TIAR (**b'**), showing a point-like signal (arrows) both in the cytoplasm and in the neuronal nucleus, partially colocalize with α-synuclein oligomers (**a", b"**). Nuclei are counterstained using Hoechst. Scale bar, 20 μm. N = Nucleus

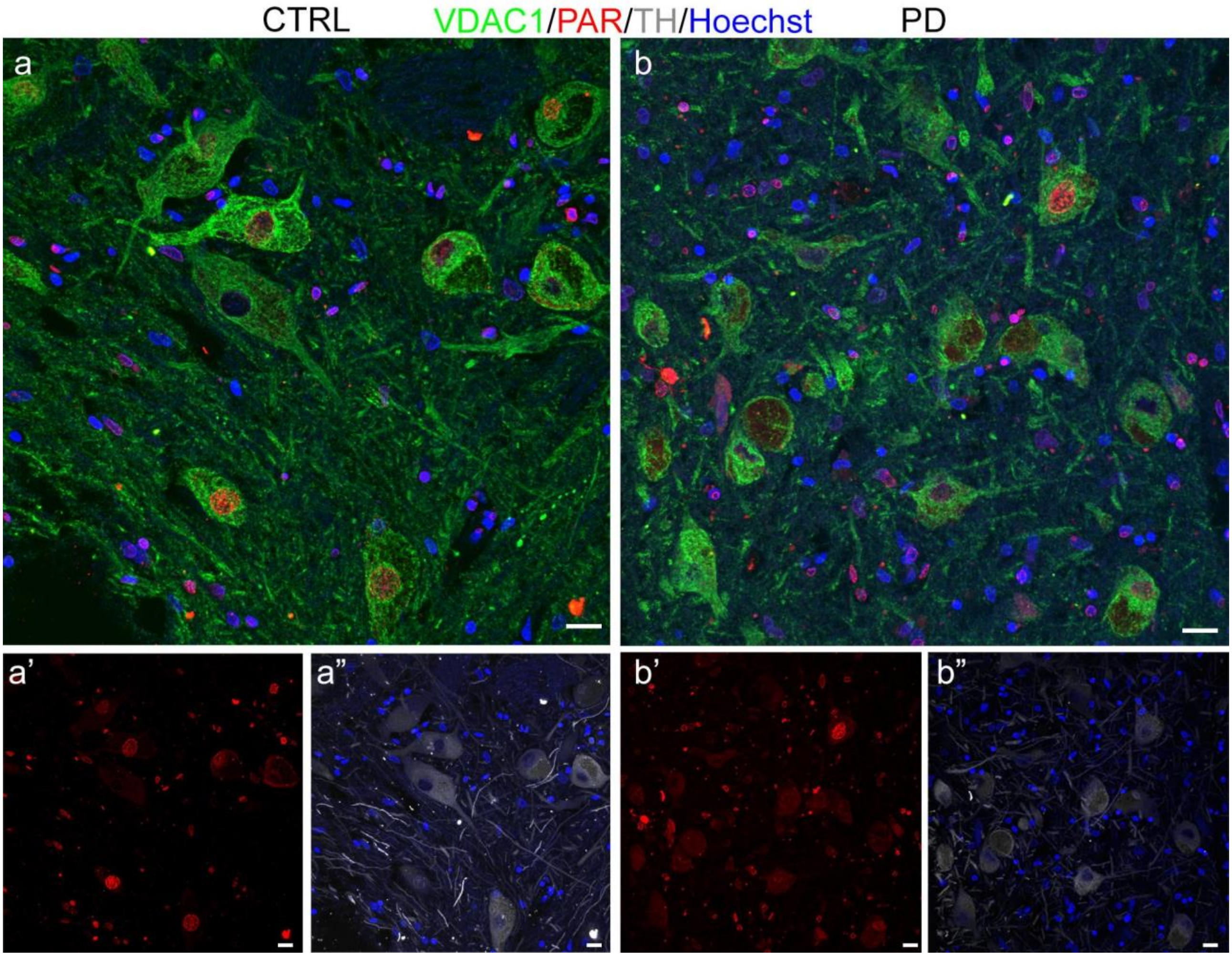

Supplementary figure 6. PAR and VDAC1 distribution in *substantia nigra* dopaminergic neurons. In dopaminergic neurons (stained with TH) of CTRL subjects, PAR is localized in the nucleus and VDAC1 staining shows the mitochondria network, as expected (a-a"). In contrast, in PD patients, VDAC1 and PAR colocalize in the cytoplasm of TH positive neurons (b-b"). Nuclei are counterstained using Hoechst. in Scale bar, 20 μm.

Supplementary Tables 1-2

Clinical Diagnosis	Gender	Age of onset	Age of death	Disease duration (year)
CTRL#1	M	/	71	/
CTRL#2	F	/	93	/
CTRL#3	F	/	82	/
CTRL#4	F	/	64	/
CTRL#5	F	/	84	/
CTRL#6	F	/	64	/
PD#1	M	62	80	18
PD#2	M	59	87	28
PD#3	F	65	84	19
PD#4	F	53	91	38
PD#5	F	59	79	20
PD#6	M	57	71	14
PD#7	M	57	75	18
PD#8	M	59	75	16

Supplementary Table 1. Demographic and clinical characteristics of the subjects included in this study.

Antigen	Code	Host	Dilution
**Primary antibodies**			
G3BP (Human G3BP1 aa 200-350)	Ab56574 Abcam	Mouse	1:250
Poly-ADP ribose (PAR)	MABC547 Merck	Mouse	1:100
S-100β (Rat S100β aa 1 to 92)	2874006 Synaptic Systems	Chicken	1:500
TIAR (Human TIAR aa 161-365)	610352 BD Transduction Laboratories™	Mouse	1:100
Tyrosine Hydroxylase (TH) (synthetic peptide sequence, VQDELDTLAHAL, corresponding to C-terminus)	PA518372 Thermo Fisher Scientific	Goat	1:200
VDAC1 (the immunogen used is proprietary information)	ab15895 Abcam	Rabbit	1:200
α-synuclein (human aa 111-132, corresponding to C-terminus)	S3062 Merck	Rabbit	1:2000
α-synuclein (human synthetic peptide aa 100 to C-terminus)	Ab21976 Abcam	Sheep	1:100
**Secondary antibodies**			

Alexa Fluor® 568 anti-mouse	A10037 Thermo Fisher Scientific	Donkey	1:200
Alexa Fluor® brilliant violet 421 anti-chicken	703-675-155 Jackson Immunoresearch	Donkey	1:300
Alexa Fluor® 647 anti-goat	705-615-147 Jackson Immunoresearch	Donkey	1:300
Alexa Fluor® 488 anti-rabbit	A21206 Thermo Fisher Scientific	Donkey	1:200
Alexa Fluor® 647 anti-rabbit	A32795 Thermo Fisher Scientific	Donkey	1:200
Alexa Fluor® brilliant violet 421 anti-sheep	713-675-147 Jackson Immunoresearch	Donkey	1:300
**Probes, kit and stains**			
Duolink® in situ probe marker MINUS	DUO920101KT Merck	-	-
Duolink® in situ probe marker PLUS	DUO920091KT Merck	-	-
Duolink® In Situ Detection Reagents green	DUO92014 Merck	-	-
EnVision FLEX DAB + Substrate Chromogen System	K3468 Agilent	-	-
Vector SG Substrate Kit, Peroxidase (HRP)	SK-4700, Vector laboratories	-	-
Hoescht 33342	62249 Thermo Fisher Scientific	-	1:5000
TO-PRO®-3	629661 Thermo Fisher Scientific	-	1:1000

Supplementary Table 2. Primary and secondary antibodies and kits used in this study.

SUPPLEMENTARY MOVIES

Movie 1: The movie refers to arivis 4D software 3D reconstruction of figure 3a’’’.

Movie 2: The movie refers to arivis 4D software 3D reconstruction of figure 3b’’’.

Movie 3: The movie refers to arivis 4D software 3D reconstruction of figure 3c’’’.
